# Health profile of older adults assisted by the Elderly Caregiver Program of Health Care Network of the City of São Paulo

**DOI:** 10.31744/einstein_journal/2020AO5263

**Published:** 2020-03-30

**Authors:** Suzana Carvalho Vaz de Andrade, Rosa Maria Bruno Marcucci, Lilian de Fátima Costa Faria, Sérgio Márcio Pacheco Paschoal, Flávio Rebustini, Ruth Caldeira de Melo

**Affiliations:** 1 Escola de Artes, Ciências e Humanidades Universidade de São Paulo São PauloSP Brazil Escola de Artes, Ciências e Humanidades, Universidade de São Paulo, São Paulo, SP, Brazil.; 2 Secretaria Municipal de Saúde São PauloSP Brazil Secretaria Municipal de Saúde, São Paulo, SP, Brazil.; 3 Faculdade de Medicina Universidade de São Paulo São PauloSP Brazil Faculdade de Medicina, Universidade de São Paulo, São Paulo, SP, Brazil.

**Keywords:** Home care services, Home health aides, Healthcare models, Health profile, Health services for the aged, Aged

## Abstract

**Objective:**

To assess the health profile of community-dwelling older adults, according to sex, assisted by the Elderly Caregiver Program of the City of São Paulo.

**Methods:**

Secondary data of 535 older adults, assisted by ten Elderly Caregiver Program teams from the southern region of São Paulo, were collected from medical records and the Multidimensional Evaluation of Older People in Primary Care, after verifying the inclusion and exclusion criteria for the study and obtaining subjects’ consent.

**Results:**

Older adults assisted by this program were predominantly female (77.6%), mean age of 76.2±8.0 years. They had negative self-rated health (67.8%), difficulties in instrumental activities of daily living (68.4%) and signs of mood changes (60.3%). A high prevalence of older adults with vision problems (58.8%), polypharmacy (58.1%), memory-related complaints (55.8%) and multiple morbidities (50.6%) were observed. The prevalence of multiple morbidities, polypharmacy, cognitive impairment and signs of mood changes were higher in women. On the other hand, men reported more hearing problems than women.

**Conclusion:**

The poorer health conditions of the older adults in this study, evidenced by a high prevalence of subjects with negative self-rated health, difficulties in instrumental activities of daily living, multiple morbidities, polypharmacy and other complaints (sensorial changes, depressive and cognitive symptoms), reinforce the importance of this program as a long-term care policy and as a way to ensure these older adults can continue living in their communities.

## INTRODUCTION

The Brazilian population ages faster compared with that of developed countries, and does so within a context of economic inequality. In 2014, older adults accounted for 13.7% of the Brazilian population, whereas projections of the Brazilian Institute of Geography and Statistics (IBGE - *Instituto Brasileiro de Geografia e Estatística*) estimate that this rate will go up to 33.7% by 2060.^(1)^ The higher number of older adults poses numerous challenges when it comes to public health, since the prevalence of non-communicable diseases (NCD), frailty and disability is higher in long-lived adults.^(2-4)^

According to the Brazilian Longitudinal Study of Aging (ELSI-Brazil - *Estudo Longitudinal de Saúde dos Idosos Brasileiros*) investigating 9,412 subjects aged over 49 and over from 70 Brazilian cities, the prevalence of multimorbidity (three or more chronic diseases) at age ranges 60-69, 70-79 and ≥80 years was 52%, 61% and 67%, respectively.^(2)^Likewise, the presence of difficulties in at least one basic activity of daily living (BADL) was higher in the oldest-old (≥80 reported 42.7%) compared with younger age groups (60-69 years reported 21.8% and 70-79 years, 26.7%), and the demand for care was higher among the oldest-old, females, and subjects with fewer years of schooling.^(4)^

Taking care of an increasingly older population is a challenge for developing countries like Brazil, which need to handle the issue of rapid population aging combined with great economic and social inequalities.^(1)^ In respect to public health policies,^(4)^ support for families with aged members has not been implemented yet throughout the territory, and policies aiming at long-term care are scarce. Currently, there are some aging in place care programs too allow aged patients to stay at home, such as the Greater Care Program in the city of Belo Horizonte,^(5)^and the Elderly Caregiver Program (PAI - *Programa Acompanhante de Idosos*) of the city of São Paulo.^(6)^

The PAI is a modality of biopsychosocial home care for older people in a situation of clinical frailty and social vulnerability, offering the services of health care professionals and professional caregivers aiming at rehabilitation, maintenance/improvement of self-care, and socialization. It was created with the purpose of providing integrated health care to dependent and socially vulnerable seniors, with difficult access to the health system and living in social isolation or exclusion, primarily due to insufficient family or social support.^(6)^ Older adults assisted by the PAI are seen by a multidisciplinary team assigned to a Primary Care Unit (UBS – *Unidade Básica de Saúde*), composed of a coordinator, a physician, a nurse, two nursing attendants/technicians, a clerk and ten professional caregivers of older adults . The common skills of PAI professionals include disease prevention and health promotion, evaluations, plans of care and home visits, as well as matrix-based actions, shared management and continued education.^(6)^

Considering the relevance of the PAI in ensuring frail and vulnerable older people can live as long as possible in their communities, we need to understand the profile of current patients to provide data for continuous improvement of the program.

## OBJECTIVE

To assess the health profile of community-dwelling older adults, according to sex, enrolled in the Elderly Care Program of the City of São Paulo.

## METHODS

### Sample

The administrative division of the Municipal Health Department of São Paulo is made up by six Regional Health Coordinations (CRS - *Coordenadorias Regionais de Saúde)* which, in turn, are subdivided into 27 Technical Health Supervisions, covering the 32 Regional Administrations within the city’s territory. This study was conducted with ten PAI teams (Parelheiros, Grajaú, Cidade Dutra, Campo Grande, Cidade Ademar, Parque Doroteia, Jardim Miriam II, Jardim Souza, Maracá and Vera Cruz), all under the CRS-South, after being locally approved by the Institutional Review Boards of the School of Arts, Sciences and Humanities of *Universidade de São Paulo* (opinion no. 2.917.083, CAAE: 92460318.4.0000.5390) and the Municipal Health Department of São Paulo (opinion no. 2.960.325, CAAE: 92460318.4.3001.0086).

The older adults assisted by the aforementioned PAI units were invited to take part in this research study, authorizing access to their medical records and their information in the Multidimensional Evaluation of Older People in Primary Care (AMPI/AB - *Avaliação Multidimensional da Pessoa Idosa na Atenção Básica*), after signing an Informed Consent Form (ICF). To be assisted by the PAI, users must be aged ≥60 years, live within the area covered by the corresponding UBS, and meet at least one of the following criteria, verified in the AMPI/AB applied by the UBS team: dependence in activities of daily living (ADL); impaired mobility; difficulty accessing health care services; insufficient family and social support; social isolation or exclusion; and being at risk for institutionalization.^(6)^

Data collection took place in November 2018, when 1,297 seniors were being assisted. This number was used for sample size calculation, and led to a minimum sample size of about 297 seniors, considering a 95% confidence interval, 5% error margin and 50% estimated rate of chronic conditions and/or health problems (*i.e*., a rate as conservative as possible, ensuring, therefore, a larger sample).

In addition to the aforementioned inclusion criteria of the PAI program per se, this study included only older adults/people who had completed the AMPI/AB. Also, the AMPI/AB had to have been applied by a trained professional at least one year before, and answered by seniors themselves, with no interferences or help from third-parties. We excluded seniors who did not authorize access to their medical records and were not able to sign the ICF due to low schooling (illiterate), cognitive impairment and/or sensory impairment. After applying the inclusion and exclusion criteria, we collected data from 535 seniors.

### Questionnaire

The AMPI/AB is a multidimensional evaluation tool used in Primary Care units in the city of São Paulo to rate the functional risk of older people. In addition to help build a registry of aged users, the AMPI/AB allows for better organization of the network’s services and preparation of plans of care; assesses seniors’ demand in the public health network, and improves the planning and management the care of the older people.^(7)^ The questionnaire covers 17 parameters (age, self-rated health, family arrangement, chronic conditions, medications, hospitalizations, falls, vision, hearing, physical limitation, cognition, mood, BADL, instrumental activities of daily living - IADL, incontinence, unintentional weight loss and oral health), based on self-reported answers to 31 questions. In addition to providing information on the needs of the older people, the AMPI/AB has also been used to rate the level of frailty and refer older adults ou older people to specialized services, if there is a verified need for therapies and/or specific care.^(6,7)^

According to AMPI/AB score, the older adults are classified as healthy (zero to 5 points), pre-frail (6 to 10 points) and frail (over 10 points). It is worth noting that this questionnaire is undergoing validation, and a preliminary study has shown good accuracy (area under the ROC - Receiver Operating Characteristic - curve - 0.851, p<0.001) in detecting frailty,^(8)^ based on the frailty phenotype proposed by Fried et al.^(9)^

### Statistical analysis

The data are presented in percentages, according to the AMPI/AB parameters and the subjects’ sex. We used the χ^2^ test to check the distribution of answers in relation to sex. For all analyses, we considered a 5% alpha.

## RESULTS

We reviewed data from 535 older adults assisted by the PAI (mean age 76.2±8.0 years). The characteristics of the older adults are shown in table 1.

**Table 1:** Characteristics of older adults assisted by the Elderly Care Program, according to sex

Parameter	Question	Answers	Total (%)	Female (%)	Male (%)	p value
			(n=535)	(n=415)	(n=120)	
1. Age	How old are you?	60-74 years	42.5	44.2	36.6	
		75-89 years	53.6	53.7	56.7	NS
		>90 years	3.9	3.1	6.7	
2. Self-rated health	Overall, compared with other people your age, would you say your health is:	Very good/good	32.2	30.3	38.3	NS
		Fair/poor/very poor	67.8	69.7	61.7	
3. Family arrangement	Do you live by yourself?	No	74.2	72.5	80.0	NS
		Yes	25.8	27.5	20.0	
4. Chronic conditions	Do you have/Have you had any of the following conditions?^*^	None	4.1	3.6	5.8	<0.01
		1 or 2	45.3	42.3	55.8	
		3 or +	50.6	54.1	38.3	
5. Medications	How many medications do you take on a daily basis?	1 to 4	41.9	37.4	57.5	<0.01
		5 or +	58.1	62.6	42.5	
6. Hospitalizations	How many times were you admitted to a hospital in the past 12 months?	None	82.7	83.3	80.8	NS
		Once	14.3	13.7	16.7	
		≥Twice	3.0	3.1	2.5	
7. Falls	How many times did you fall in the last 12 months?	None	61.2	61.0	62.2	NS
		Once	20.5	19.9	22.7	
		≥Twice	18.2	19.1	15.1	
8. Vision	Do you have any difficulty seeing? (even with glasses)	No	41.2	41.4	40.7	NS
		Yes	58.8	58.6	59.3	
9. Hearing	Do you have any difficulty hearing or do people think your hearing is poor?	No	58.9	62.8	45.2	<0.01
		Yes	41.1	37.2	54.6	
10. Physical limitation	Does the subject have any physical limitation?^†^	No	53.3	48.9	60.8	NS
		Yes	46.7	51.1	39.2	
11. Cognition	Does the subject have any sign of cognitive impairment?^‡^	No	44.2	41.8	52.5	<0.05
		Yes	55.8	58.2	47.5	
12. Mood	Does the subject show any mood changes?^§^	No	39.7	36.0	52.5	<0.01
		Yes	60.3	64.0	47.5	
13. BADL	Does the subject need help with any BADL?^¶^	No	82.6	83.8	78.3	NS
		Yes	17.4	16.2	21.7	
14. IADL	Does the subject need help with any IADL?^&^	No	31.6	30.1	36.7	NS
		Yes	68.4	69.9	63.3	
15. Incontinence	Does the subject have any sign of incontinence?^#^	No	58.7	58.2	60.5	NS
		Yes	41.3	41.8	39.5	
16. Unintentional weight loss	In the last 12 months, did you lose weight without dieting or changing your lifestyle habits?	No	74.3	73.2	78.0	NS
		Yes	25.7	26.8	22.0	
17. Oral health	Does the subject have any oral health or food intake problems?**	No	48.7	47.5	52.9	NS
		Yes	51.3	52.5	47.1	

^*^ Referring to the following conditions: *diabetes mellitus*, hypertension, stroke, coronary artery disease, vascular diseases, pressure ulcers, anemia, asthma, chronic obstructive pulmonary disease, peptic ulcer, osteoarthritis, obesity, cancer, dementia, epilepsy, depression, Parkinson´s disease, HIV/AIDS, limb amputation, smoking and alcohol use; ^†^ abilities assessed: touching the back of the neck, lifting a pencil from a table with one hand and putting it back, walking 400m, and sitting or standing up with no difficulty; ^‡^ signs assessed: other people claiming the subject is increasingly forgetful, aggravation of forgetfulness in recent months, forgetfulness is preventing the subject from performing any activity of daily living; ^§^ signs present in the last month: lack of drive, sadness or hopelessness, loss of interest or pleasure in activities previously enjoyable; ^¶^ activities assessed: getting out of bed, getting dressed, eating and bathing; ^&^ activities assessed: out-of-home activities and money management; ^#^ incontinence: involuntary loss of urine and feces, ^**^ conditions assessed: poorly adapted dentures, difficulty chewing, difficulty swallowing, not eating food due to dental problems/dentures. NS: non-significant; BADL: basic activities of daily living; IADL: instrumental activities of daily living.

A little over half of the population assisted by the PAI at CRS-South was in the 75-89-year age group (53.6%), followed by 60-74-years (42.5%). The rate of oldest-old subjects (aged ≥90) was only 3.9%.

As for sex, there were more females (77.6%). Among the issues assessed by the AMPI/AB, the following stand out negative self-rated health (67.8% reported having fair, poor or very poor health), presence of multiple morbidities (50.6% reported having three or more chronic diseases), polypharmacy (58.1% used five or more medications), presence of sensory problems (58.8% had problems seeing and 41.1% hearing), complaints of cognitive impairment (55.8% had signs of cognitive deficit), mood changes (60.3% had depressive symptoms), difficulty performing IADL (68.4% required help in at least one activity), and presence of urinary incontinence and/or fecal incontinence (41.3%). Oral health or food intake problems were also reported by half of the older adults (51.3%), whereas only 25.7% reported having lost weight for no apparent reason in the last 12 months.

As for family arrangement, 25.8% of respondents (total sample) declared to live by themselves. The prevalence of recurrent falls and hospitalizations (two or more in the past year) was 18.2 and 3.0%, respectively. Most of the older adults reported no functional limitations preventing them from performing self-care activities (82.6%), although 46.7% reported having some physical limitation.

Between the sexes, we found that a higher proportion of women had three or more chronic diseases (54.1% *versus* 38.3%), signs of cognitive impairment (58.2% *versus* 47.5%), mood changes (64.0% *versus* 47.5%) and polypharmacy (62.6% *versus* 42.5%) compared with men (p<0.05). The other parameters (age, self-rated health, family arrangement, hospitalizations, falls, vision, physical limitation, BADL, IADL, incontinence, unintentional weight loss and oral health conditions) showed no differences between the sexes. However, hearing impairment was more frequent among men (54.6%) than women (37.2).

**Figure 1:**
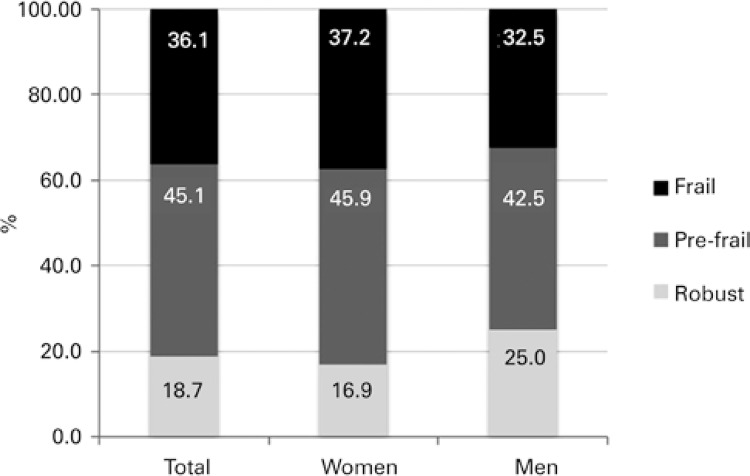
Prevalence of frailty in older adults assisted by the Elderly Caregiver Program, based on the final score of the Multidimensional Evaluation of Older People in Primary Care AMPI/AB: Multidimensional Evaluation of Older People in Primary Care.

With respect to the final classification in the AMPI/AB, the majority of the population assisted by the PAI was classified as pre-frail (45.1%) and frail (36.1%), and no statistical differences were observed in the prevalence of frailty between the sexes (Figure 1).

## DISCUSSION

The prevalence of females assisted by the PAI was slightly higher (77.6%) than in other national studies, such as that conducted by the Brazilian Frailty in Older People (FIBRA - *Fragilidade em Idosos Brasileiros*) network,^(10)^ which interviewed 5,532 seniors from different regions of Brazil, with a prevalence of females of 65.6%. Similarly, a study with data from older people (60 and older) enrolled in the ELSI-Brazil,^(11)^ the prevalence of females was 53.6%.^(3)^

The feminization of old age, *i.e.*, the greater proportion of women than man in the older population, particularly among the oldest-old, has relevant repercussions on the need for public policies. Compared with men, women tend to live longer with NCD and are at higher risk for dependence, in addition to commonly undertaking the role of caregivers for their spouses and then being alone at the end of life.^(4,12)^ Since women also have lower schooling and wages when compared to men, they are more susceptible to frailty and lack of financial resources in old age.^(12,13)^ Therefore, women have a greater chance of needing long-term care, due to greater disability as well as low social support, which could explain the greater proportion of older women assisted by the PAI.

This change in the health profile of the population of developing countries is taking place fast, and NCD now feature among the major causes of death and disability, in positions that were formerly occupied by infectious-contagious diseases.^(1)^ Although aging is not synonymous with disease, the literature supports that NCD are more prevalent as we get older.^(14)^ This is a relevant fact which impacts public policies, considering that NCD aggravate health conditions and may lead to functional decline, low quality of life, increased use of health care services, and higher health care costs.^(14)^

In Brazilian community-dwelling older adults, the prevalence of multiple morbidities (two or more chronic diseases) varies from 36% to 42%.^(2,13)^ In PAI subjects, however, the prevalence of three or more chronic diseases was 50.6%, and even higher among females (54.1%). Data from the Health, Well-Being and Aging study (SABE - *Saúde, Bem-Estar e Envelhecimento*) also confirm this greater prevalence of NCD in older women. Alvarado et al.,^(13)^ showed that 45.4% of older women enrolled in the SABE-Brazil study reported having two or more chronic diseases, whereas for men, the rate was 36.4%.

Another very common problem in aged patients with multiple morbidities is the excessive and/or improper use of medications. Population-based studies show that the prevalence of polypharmacy (regular use of five or more medications) among older adults may vary between 10.3% and 36%,^(15-18)^which is lower than what was found in this study (58.1%). The main factors related with polypharmacy include negative self-rated health, the presence of NCD (especially cardiovascular and metabolic), and the use of health care services.^(16-18)^ Additionally, data from the SABE-Brazil study^(15)^ showed a greater risk of polypharmacy among women, and the oldest-old (aged >75 years). Among the older adults in this study, the majority were female (77.6%) and a large part was aged ≥75 years (57.5%), which may have contributed to the high rate of polypharmacy. We also found a greater prevalence of polypharmacy among females (62.6%) compared to males (42.5%). Polypharmacy increases the risk of negative outcomes in the old age, such as adverse reactions, functional decline, cognitive decline, urinary incontinence, falls, among others,^(19)^and this practice is considered a challenge for elderly care.^(16,17)^

Self-rated health has been associated with the risk of death, particularly in the older adults.^(20-22)^ According with Reile et al.,^(20)^subjects with negative self-rated health have a twice-as-high risk of mortality in 5 years, compared with those who self-rate their health as excellent, even after adjusting for relevant covariates (comorbidities, functional capacity and depression). Bamia et al.,^(21)^ also found an increased risk of mortality in aged patients with negative self-rated health in different regions of Europe and the United States, and therefore concluded this is a quick and simple tool to identify groups of older people at risk for early mortality.

According to Kusumastuti et al.,^(22)^the possibility to determine someone’s risk of death using this health self-assessment is the same as with other more objective scores (comorbidity and frailty scores), which highlights the importance of considering the older adults self-perception of their own health status.

In this study, 67.8% of PAI participants reported having fair, poor or very poor health, which was higher than found in population-based data of community-dwelling older adults. Data from the last wave of the SABE-Brazil study, for example, showed that 48.9% of respondents self-rated their health as fair, poor or very poor,^(23,24)^ and this was associated with the female sex, age ≥75 years, low income and fewer years of schooling.

According to the World Health Organization (WHO), the health of older people must be considered from a functional perspective, not based on the absence of disease.^(25)^Functionality, in turn, is determined by the level of help required by subjects for ADL, which is influenced by personal (physical and mental skills) and environmental factors, and the interaction between them.^(26)^ The establishment of disability follows a hierarchical process that typically starts with the onset of a morbidity, leading to dysfunction, followed by functional limitations and, ultimately, full-blown disability.^(27,28)^ According to Bleijenber et al.,^(29)^older people with three or more chronic diseases have a three-to-five-fold higher risk of disability, as compared to individuals not suffering from such diseases. Therefore, it is essential to intervene early in the process of disability, with the view to preserve as much as possible the functional capacity of older people at risk.

In respect to ADL difficulties, we found that 17.4% of older adults enrolled in the PAI require help for at least one BADL. This finding supports other studies with Brazilian older adults, with the prevalence of BADL difficulties in community-dwelling older adults varying between 14.8% and 25.4%.^(4)^ However, the rate of PAI participants who require help for IADL is twice as high (68.4%) compared with the SABE-Brazil study (approximately 30%).^(4)^ This points to a greater level of dependence among PAI subjects, particularly in more complex tasks that are critical for maintaining their independence in the community. This raises concern, since the ability to perform complex activities seems to decline faster than for basic activities, and this can rapidly increase the level of dependence of these older adults.^(30)^

By considering functionality as the main determinant of health in old age, it is imperative that older people’s care be based on managing functional capacity and not existing chronic conditions.^(31)^ Thus, in the line of care proposed for the older adults, the risk of developing frailty must be monitored based on the functional capacity and, as a consequence, and according to the needs, at different levels of care.^(32)^ In the home care setting, the PAI contributes with integrated care for older adults in a situation of frailty and vulnerability, by providing support with health and social needs.^(33)^ In this sense, the different health-related demands found among older adults enrolled in this study reinforce the major role of the PAI in maintaining the independence and autonomy of its users.

Finally, this study has some limitations that should be considered. By excluding illiterate older adults and those with cognitive and sensory impairment, due to their difficulty answering the questions in the AMPI/AB and/or signing the informed consent form, we have potentially excluded the most frail and vulnerable older people from the final sample. In addition, since the AMPI/AB is under validation, the authors chose not to discuss any data relative to the frailty classification, which still needs to be tested and confirmed in future psychometric studies.

## CONCLUSION

The poorer health conditions of vulnerable older adults assisted by the Elderly Caregiver Program, proven by the high prevalence of negative self-rated health, difficulties in instrumental activities of daily living, multiple morbidities, polypharmacy, and other complaints (sensory impairment, depressive and cognitive symptoms) underline the importance of this program as a long-term care policy, helping, therefore, ensure these seniors can continue living for as long as possible in their communities.

## References

[1] 1. Tramujas Vasconcellos Neumann L, Albert SM. Aging in Brazil. Gerontologist. 2018;58(4):611-7. 10.1093/geront/gny019 30010820

[2] 2. Nunes BP, Batista SR, Andrade FB, Souza Junior PB, Lima-Costa MF, Facchini LA. Multimorbidity: The Brazilian Longitudinal Study of Aging (ELSI-Brazil). Rev Saude Publica. 2018;52(Suppl 2):10s. 10.11606/S1518-8787.2018052000637 PMC6254906 30379288

[3] 3. Andrade JM, Duarte YA, Alves LC, Andrade FC, Souza Junior PR, Lima-Costa MF, et al. Frailty profile in Brazilian older adults: ELSI-Brazil. Rev Saude Publica. 2018;52(Suppl 2):17s. 10.11606/S1518-8787.2018052000616 PMC6255048 30379282

[4] 4. Giacomin KC, Duarte YA, Camarano AA, Nunes DP, Fernandes D. Care and functional disabilities in daily activities – ELSI-Brazil. Rev Saude Publica. 2018;52(Suppl 2):9s. 10.11606/S1518-8787.2018052000650 PMC6254988 30379293

[5] 5. Alcântara AD, Camarano AA, Giacomin KC. Política Nacional do Idoso: velhas e novas questões. Rio de Janeiro: IPEA; 2016.

[6] 6. Secretaria Municipal da Saúde. Coordenação da Atenção Básica. Área Técnica de Saúde da Pessoa Idosa. Programa Acompanhante de Idosos. Documento Norteador Programa Acompanhante de Idosos [Internet]. São Paulo: SMS; 2016 [citado 2019 Jul 16]. Disponível em: https://www.prefeitura.sp.gov.br/cidade/secretarias/upload/saude/DOCUMENTONORTEADORPAIFINAL02012017.pdf

[7] 7. Secretaria Municipal da Saúde. Coordenação da Atenção Básica. Área Técnica de Saúde da Pessoa Idosa. Documento Norteador Unidade de Referência à Saúde do Idoso URSI [Internet]. São Paulo: SMS; 2016 [citado em em 2019 Set 7]. Disponível em: https://www.prefeitura.sp.gov.br/cidade/secretarias/upload/saude/arquivos/ANEXOSDOCNORTURSI21122016.pdf

[8] 8. American Geriatrics Society 2019 Annual Scientific Meeting. AMPI-AB accuracy: a multidimensional questionnaire for the management of the public healthcare for older people in the city of São Paulo, Brazil [Internet]. Portland, OR; May 2 – 4, 2019. p. S47 [cited 2020 Mar 5]. Available from: https://meeting.americangeriatrics.org/sites/default/files/inline-files/2019%20JAGS%20abstract%20supplement.pdf

[9] 9. Fried LP, Tangen CM, Walston J, Newman AB, Hirsch C, Gottdiener J, Seeman T, Tracy R, Kop WJ, Burke G, McBurnie MA; Cardiovascular Health Study Collaborative Research Group. Frailty in older adults: evidence for a phenotype. J Gerontol A Biol Sci Med Sci. 2001;56(3):M146-56. 10.1093/gerona/56.3.m146 11253156

[10] 10. Silva SL, Neri AL, Ferrioli E, Lourenço RA, Dias RC. [Phenotype of frailty: the influence of each item in determining frailty in community-dwelling elderly - The Fibra Study]. Cienc Saude Coletiva. 2016;21(11):3483-92. 10.1590/1413-812320152111.23292015 27828581

[11] 11. Lima-Costa MF, de Andrade FB, de Souza PR Jr, Neri AL, Duarte YA, Castro-Costa E, et al. The Brazilian Longitudinal Study of Aging (ELSI-Brazil): Objectives and Design. Am J Epidemiol. 2018;187(7):1345–53. 10.1093/aje/kwx387 PMC6031009 29394304

[12] 12. Muñoz Cobos F, Espinosa Almendro JM. [Active ageing and gender inequalities]. Aten Primaria. 2008;40(6):305-9. Spanish. 10.1157/13123684 PMC7713279 18588803

[13] 13. Alvarado BE, Zunzunegui MV, Béland F, Bamvita JM. Life course social and health conditions linked to frailty in Latin American older men and women. J Gerontol A Biol Sci Med Sci. 2008;63(12):1399-406. 10.1093/gerona/63.12.1399 19126855

[14] 14. Marengoni A, Angleman S, Melis R, Mangialasche F, Karp A, Garmen A, et al. Aging with multimorbidity: a systematic review of the literature. Ageing Res Rev. 2011;10(4):430-9. Review. 10.1016/j.arr.2011.03.003 21402176

[15] 15. Carvalho MF, Romano-Lieber NS, Bergsten-Mendes G, Secoli SR, Ribeiro E, Lebrão ML, et al. Polypharmacy among the elderly in the city of São Paulo, Brazil - SABE Study. Rev Bras Epidemiol. 2012;15(4):817-27. 10.1590/s1415-790x2012000400013 23515777

[16] 16. Ramos LR, Tavares NU, Bertoldi AD, Farias MR, Oliveira MA, Luiza VL, et al. Polypharmacy and Polymorbidity in Older Adults in Brazil: a public health challenge. Rev Saude Publica. 2016;50(Suppl 2):9s. 10.1590/S1518-8787.2016050006145 PMC5157903 27982377

[17] 17. Nascimento RC, Álvares J, Guerra AA Junior, Gomes IC, Silveira MR, Costa EA, et al. Polypharmacy: a challenge for the primary health care of the Brazilian Unified Health System. Rev Saude Publica. 2017;51(Suppl 2):19s. 10.11606/S1518-8787.2017051007136 PMC5676396 29160460

[18] 18. Almeida NA, Reiners AA, Azevedo RC, Silva AM, Cardoso JD, Souza LC. Prevalence of and factors associated with polypharmacy among elderly persons resident in the community. Rev Bras Geriatr Gerontol. 2017;20(1):138-48.

[19] 19. Maher RL, Hanlon JT, Hajjar ER. Clinical consequences of polypharmacy in elderly. Expert Opin Drug Saf. 2014;13(1):57-65. Review. 10.1517/14740338.2013.827660 PMC3864987 24073682

[20] 20. Reile R, Stickley A, Leinsalu M. Re: Letter to the Editor of Public Health in response to ‘Large variation in predictors of mortality by levels of self-rated health: results from an 18-year follow-up study’. Public Health. 2017; 147:157-8. 10.1016/j.puhe.2017.03.017 28476204

[21] 21. Bamia C, Orfanos P, Juerges H, Schöttker B, Brenner H, Lorbeer R, et al. Self-rated health and all-cause and cause-specific mortality of older adults: Individual data meta-analysis of prospective cohort studies in the CHANCES Consortium. Maturitas. 2017;103:37-44. 10.1016/j.maturitas.2017.06.023 28778331

[22] 22. Kusumastuti S, Gerds TA, Lund R, Mortensen EL, Westendorp RG. Discrimination ability of comorbidity, frailty, and subjective health to predict mortality in community-dwelling older people: Population based prospective cohort study. Eur J Intern Med. 2017;42:29-38. 10.1016/j.ejim.2017.05.016 28583408

[23] 23. DeSalvo KB, Bloser N, Reynolds K, He J, Muntner P. Mortality prediction with a single general self-rated health question. A meta-analysis. J Gen Intern Med. 2006;21(3):267-75. 10.1111/j.1525-1497.2005.00291.x PMC1828094 16336622

[24] 24. Antunes JL, Chiavegatto Filho AD, Duarte YA, Lebrão ML. Social inequalities in the self-rated health of the elderly people in the city of São Paulo, Brazil. Rev Brasil Epidemiol. 2019;21(Suppl 2):e180010. 10.1590/1980-549720180010.supl.2 30726355

[25] 25. World Health Organization (WHO). Ageing and life-course. World report on ageing and health 2015 [Internet]. Geneva: WHO; 2015 [cited 2019 Jul 16]. Available from: https://www.who.int/ageing/events/world-report-2015-launch/en/

[26] 26. Cesari M, Araujo de Carvalho I, Amuthavalli Thiyagarajan J, Cooper C, Martin FC, Reginster JY, et al. Evidence for the Domains Supporting the Construct of Intrinsic Capacity. J Gerontol A Biol Sci Med Sci. 2018;73(12):1653-60. Review. 10.1093/gerona/gly011 29408961

[27] 27. Verbrugge LM, Jette AM. The disablement process. Soc Sci Med. 1994; 38(1):1-14. 10.1016/0277-9536(94)90294-1 8146699

[28] 28. Gu D, Gomez-Redondo R, Dupre ME. Studying disability trends in aging populations. J Cross Cult Gerontol. 2015;30(1):21-49. Review. 10.1007/s10823-014-9245-6 25391217

[29] 29. Bleijenberg N, Zuithoff NP, Smith AK, de Wit NJ, Schuurmans MJ. Disability in the individual ADL, IADL, and mobility among older adults: a prospective cohort study. J Nutr Health Aging. 2017;21(8):897-903.10.1007/s12603-017-0891-628972242

[30] 30. Bendayan R, Cooper R, Wloch EG, Hofer SM, Piccinin AM, Muniz-Terrera G. Hierarchy and speed of loss in physical functioning: a comparison across older U.S. and english men and women. J Gerontol A Biol Sci Med Sci. 2017;72(8):1117-22. 10.1093/gerona/glw209 PMC5861940 27753610

[31] 31. Veras RP, Caldas CP, Cordeiro HA. Modelos de atenção à saúde do idoso: repensando o sentido da prevenção. Physis Rev Saude Coletiva. 2013;23(4):1189-213.

[32] 32. Veras RP, Caldas CP, Cordeiro HA, Motta LB, Lima KC. Desenvolvimento de uma linha de cuidados para o idoso: hierarquização da atenção baseada na capacidade funcional. Rev Bras Geriatr Gerontol. 2013;16(2):385-92.

[33] 33. Ferreira FC, Bansi LO, Paschoal SP. Serviços de atenção ao idoso e estratégias de cuidado domiciliares e institucionais. Rev Bras Geriatr Gerontol. 2014; 17(4):911-26.

