# Supplementary material for: Health profile of older adults assisted by the Elderly Caregiver Program of Health Care Network of the City of São Paulo
Source: Einstein (Sao Paulo). 2020 Mar 30;18:eAO5263. doi: 10.31744/einstein_journal/2020AO5263 (PMC7108816; doi:10.31744/einstein_journal/2020AO5263)
Supplement: Translation

# Translation: Perfil de saúde dos idosos assistidos pelo Programa Acompanhante de Idosos na Rede de Atenção à Saúde do Município de São Paulo

**Authors:** Suzana Carvalho Vaz de Andrade, Rosa Maria Bruno Marcucci, Lilian de Fátima Costa Faria, Sérgio Márcio Pacheco Paschoal, Flávio Rebustini, Ruth Caldeira de Melo

**Affiliations:** 1 Brasil Escola de Artes, Ciências e Humanidades, Universidade de São Paulo, São Paulo, SP, Brasil.; 2 Brasil Secretaria Municipal de Saúde, São Paulo, SP, Brasil.; 3 Brasil Faculdade de Medicina, Universidade de São Paulo, São Paulo, SP, Brasil.

**Keywords:** Serviços de assistência domiciliar, Visitadores domiciliares, Modelos de assistência à saúde, Perfil de saúde, Serviços de saúde para idosos, Idoso

## Abstract

**Objetivo:**

Analisar o perfil de saúde de idosos residentes na comunidade, de acordo com o sexo, cadastrados no Programa Acompanhante de Idosos do Município de São Paulo.

**Métodos:**

Os dados secundários de 535 idosos assistidos por dez equipes do Programa Acompanhante de Idosos da região sul de São Paulo foram coletados a partir da consulta em prontuários e da Avaliação Multidimensional da Pessoa Idosa na Atenção Primária, após verificados os critérios de inclusão e exclusão para o estudo e a autorização dos idosos selecionados.

**Resultados:**

Os idosos assistidos por este programa eram predominantemente do sexo feminino (77,6%), apresentavam média de idade 76,2±8,0 anos, tinham autoavaliação negativa de saúde (67,8%), comprometimento das atividades instrumentais da vida diária (68,4%) e indícios de alterações de humor (60,3%). Foi observada alta prevalência de idosos com dificuldades para enxergar (58,8%), polifarmácia (58,1%), queixas de problemas de memória (55,8%) e com múltiplas morbidades (50,6%). Na comparação entre os sexos, a presença de múltiplas morbidades, polifarmácia, queixas cognitivas e sinais de alterações de humor foi maior entre as mulheres. Por outro lado, a demanda de maior prevalência entre os homens foi referente a dificuldades em ouvir.

**Conclusão:**

As piores condições de saúde desses idosos, constatadas pela alta prevalência de autoavaliação negativa de saúde, pelo comprometimento das atividades instrumentais da vida diária, por múltiplas morbidades, pela polifarmácia e outras queixas (alterações sensoriais, sintomas depressivos e alterações cognitivas), reafirmam a importância deste programa como política de cuidados de longa duração e para a manutenção destes idosos residindo na comunidade.

## INTRODUÇÃO

A população brasileira envelhece de maneira mais rápida comparada aos países desenvolvidos e em um contexto de desigualdade socioeconômica. Em 2014, os idosos representavam 13,7% da população brasileira, enquanto projeções do Instituto Brasileiro de Geografia e Estatística (IBGE) estimam que esta proporção suba para 33,7% em 2060.^(1)^ O aumento do número de idosos traz diversos desafios em termos de saúde pública, uma vez que a prevalência de doenças crônicas não transmissíveis (DCNT), fragilidade e incapacidades é maior nos mais longevos.^(2-4)^

De acordo o estudo “Estudo Longitudinal de Saúde dos Idosos Brasileiros (ELSI-Brasil)”, o qual avaliou 9.412 indivíduos com idade acima de 49 anos, e residentes em 70 cidades do Brasil, a prevalência de multimorbidades (três ou mais doenças crônicas) nas faixas etárias de 60 a 69, 70 a 79 e ≥80 anos foi de, respectivamente, 52%, 61% e 67%.^(2)^Da mesma forma, a presença de dificuldades em pelo menos uma atividades básicas da vida diária (ABVD) foi maior nos mais velhos (≥80 anos registraram 42,7%), comparativamente com as faixas etárias mais jovens (60 a 69 anos registraram 21,8% e 70 a 79 anos registraram 26,7%), sendo a demanda por cuidados maior nos indivíduos mais velhos, do sexo feminino e com menor escolaridade.^(4)^

O cuidado de uma população cada vez mais velha é um desafio para os países em desenvolvimento, como o Brasil, que precisam lidar com o rápido envelhecimento populacional em conjunto com grandes desigualdades econômicas e sociais.^(1)^ Dentro das políticas públicas de saúde,^(4)^ a abordagem da família com pessoas idosas ainda não está implementada em todo o território nacional, sendo escassas as políticas destinadas aos cuidados de longa duração. Atualmente, há alguns programas de assistência ao idoso voltados à garantia de permanência na própria residência como, por exemplo, o Programa Maior Cuidado, do munícipio de Belo Horizonte,^(5)^ e o Programa Acompanhante de Idosos (PAI), do município de São Paulo.^(6)^

O PAI é uma modalidade de cuidado domiciliar biopsicossocial à pessoa idosa em situação de fragilidade clínica e vulnerabilidade social, que disponibiliza a prestação dos serviços de profissionais da saúde e acompanhantes de idosos, visando à reabilitação, à manutenção/desenvolvimento do autocuidado e à socialização. Foi criado com o objetivo de prover assistência integral à saúde de idosos dependentes e socialmente vulneráveis, com dificuldades de acesso ao sistema de saúde e isolamento ou exclusão social, principalmente devido à insuficiência do suporte familiar ou social.^(6)^ Os idosos assistidos pelo PAI são acompanhados por uma equipe multidisciplinar, alocada em uma Unidade Básica de Saúde (UBS), composta por um coordenador, um médico, um enfermeiro, dois auxiliares/técnicos de enfermagem, um auxiliar administrativo e dez acompanhantes de idosos. Dentre as competências comuns aos profissionais do PAI, destacam-se ações de prevenção de agravos e promoção à saúde; a realização de avaliações, planos de cuidados e visitas domiciliares; e as ações de matriciamento, gestão compartilhada e educação permanente.^(6)^

Considerando a importância do PAI para a manutenção dos idosos frágeis e vulneráveis vivendo por mais tempo na comunidade, faz-se necessário conhecer o perfil daqueles atualmente assistidos, com o intuito de fornecer dados para a melhoria contínua deste programa.

## OBJETIVO

Analisar o perfil de saúde de idosos residentes na comunidade, de acordo com o sexo, cadastrados no Programa Acompanhante de Idosos do Município de São Paulo.

## MÉTODOS

### Amostra

A divisão administrativa da Secretaria Municipal de Saúde de São Paulo está distribuída em seis Coordenadorias Regionais de Saúde (CRS) que, por sua vez, se dividem em 27 Supervisões Técnicas de Saúde, as quais dão cobertura às 32 Prefeituras Regionais que compõem o território da cidade. O presente estudo foi realizado junto a dez equipes do PAI (Parelheiros, Grajaú, Cidade Dutra, Campo Grande, Cidade Ademar, Parque Doroteia, Jardim Miriam II, Jardim Souza, Maracá e Vera Cruz), todas pertencentes ao território da CRS-Sul, após o mesmo ter sido autorizado localmente e aprovado pelos Comitês de Ética em Pesquisa em Seres Humanos da Escola de Artes, Ciências e Humanidades da Universidade de São Paulo (parecer número: 2.917.083, CAAE: 92460318.4.0000.5390) e da Secretaria Municipal de Saúde de São Paulo (parecer número: 2.960.325, CAAE: 92460318.4.3001.0086).

Os idosos acompanhados pelas unidades do PAI citadas anteriormente foram convidados a participar da pesquisa, autorizando o acesso ao prontuário e às informações contidas na Avaliação Multidimensional da Pessoa Idosa na Atenção Básica (AMPI/AB), mediante assinatura do Termo de Consentimento Livre e Esclarecido (TCLE). Para ser acompanhado pelo PAI, o usuário deve ter idade ≥60 anos, residir na área de abrangência da UBS e apresentar pelo menos um dos seguintes critérios, detectados a partir da AMPI/AB realizada pela UBS: dependência nas atividades da vida diária (AVD); mobilidade reduzida; dificuldade de acesso aos serviços de saúde; insuficiência no suporte familiar e social; isolamento ou exclusão social; e risco de institucionalização.^(6)^

A coleta de dados ocorreu em novembro de 2018, quando havia 1.297 idosos em acompanhamento. Esse número foi utilizado para o cálculo amostral, que indicou a necessidade de se alcançar um tamanho de amostra mínimo da ordem de 297 idosos, considerando intervalo de confiança de 95%, margem de erro de 5% e frequência estimada de condições crônicas e/ou problemas de saúde de 50% (ou seja, frequência mais conservadora possível e, portanto, que garante maior amostra).

Além dos critérios de inclusão do próprio PAI descritos acima, foram incluídos no estudo apenas os idosos que possuíam AMPI/AB completa. Adicionalmente, era necessário que as AMPI/AB tivessem sido aplicadas por profissionais capacitados há pelo menos 1 ano e respondidas pelos próprios idosos sem interferências ou respostas de terceiros. Foram excluídos do estudo os idosos que não permitiram a consulta dos prontuários e não conseguiram assinar o TCLE devido a baixa escolaridade (analfabetos), alterações cognitivas e/ou alterações sensoriais. Após a aplicação dos critérios de inclusão e de exclusão, os dados de 535 idosos foram coletados.

### Instrumento

A AMPI/AB é um instrumento de avaliação multidimensional utilizado na Atenção Primária de Saúde do Município de São Paulo para classificar o risco funcional das pessoas idosas. Além de auxiliar na construção do cadastro dos usuários idosos, a AMPI/AB favorece a organização do atendimento na rede e a elaboração de planos de cuidados; qualifica a demanda de idosos na rede pública e visa ao planejamento e à gestão do cuidado da pessoa idosa.^(7)^ Esse instrumento avalia 17 parâmetros (idade, autoavaliação de saúde, arranjo familiar, doenças crônicas, medicamentos, internações, quedas, visão, audição, limitação física, cognição, humor, ABVD, atividades instrumentais de vida diária − (AIVD), incontinência, perda de peso não intencional e condições bucais) determinados a partir de 31 questões autorreferidas. Além de oferecer informações sobre as demandas dos idosos, a AMPI/AB tem sido utilizada para classificar o grau de fragilidade e encaminhar os idosos para serviços de atenção especializada, caso seja detectada necessidade de tratamentos e/ou acompanhamentos específicos.^(6,7)^

De acordo com a pontuação na AMPI/AB, os idosos são classificados em saudáveis (zero a 5 pontos), pré-frágeis (6 a 10 pontos) e frágeis (superior a 10 pontos). Vale ressaltar que esse instrumento está em processo de validação, e um estudo preliminar mostrou boa acurácia (área da curva ROC − de 0,851, p<0,001) para a determinação de fragilidade,^(8)^ com base no fenótipo de fragilidade proposto por Fried et al.^(9)^

### Análise estatística

Os dados estão apresentados em percentagem, de acordo com os parâmetros da AMPI/AB e com o sexo dos participantes. Para a comparação da distribuição das respostas em relação ao sexo, utilizou-se o teste χ^2^. Para todas as análises, considerou-se o alfa de 5%.

## RESULTADOS

Foram analisados os dados de 535 idosos assistidos pelo PAI (média de idade de 76,2±8,0 anos). As características dos idosos estão apresentadas na tabela 1.

**Tabela 1:** Características dos idosos assistidos pelo Programa Acompanhante de Idosos, de acordo com o sexo

Parâmetro	Pergunta	Respostas	Total (%) (n=535)	Feminino (%) (n=415)	Masculino (%) (n=120)	Valor de p
1. Idade	Qual sua idade?	60-74 anos	42,5	44,2	36,6	
		75-89 anos	53,6	53,7	56,7	NS
		>90 anos	3,9	3,1	6,7	
2. Autopercepção de saúde	Em geral, comparado com outras pessoas de sua idade, o(a) Sr.(a.) diria que sua saúde é:	Muito boa/boa	32,2	30,3	38,3	NS
		Regular/ruim/ muito ruim	67,8	69,7	61,7	
3. Arranjo familiar	O(a) Sr.(a.) mora sozinho?	Não	74,2	72,5	80,0	NS
		Sim	25,8	27,5	20,0	
4. Condições crônicas	O(a) Sr.(a.) teve/tem algumas dessas condições abaixo?^*^	Nenhuma	4,1	3,6	5,8	<0,01
		1 ou 2	45,3	42,3	55,8	
		3 ou +	50,6	54,1	38,3	
5. Medicamentos	Quantos medicamentos o(a) Sr.(a.) toma diariamente?	1 a 4	41,9	37,4	57,5	<0,01
		5 ou +	58,1	62,6	42,5	
6. Internações	Quantas vezes o(a) Sr.(a.) ficou internado(a) nos últimos 12 meses?	Nenhuma	82,7	83,3	80,8	NS
		1 vez	14,3	13,7	16,7	
		*≥*2 vezes	3,0	3,1	2,5	
7. Quedas	Quantas vezes o(a) Sr.(a.) caiu nos últimos 12 meses?	Nenhuma	61,2	61,0	62,2	NS
		1 vez	20,5	19,9	22,7	
		*≥*2 vezes	18,2	19,1	15,1	
8. Visão	O(a) Sr.(a.) tem alguma dificuldade para enxergar? (mesmo usando óculos)	Não	41,2	41,4	40,7	NS
		Sim	58,8	58,6	59,3	
9. Audição	O(a) Sr.(a.) tem alguma dificuldade para ouvir ou as pessoas acham que o(a) senhor(a) ouve mal?	Não	58,9	62,8	45,2	<0,01
		Sim	41,1	37,2	54,6	
10. Limitação física	O idoso apresenta alguma limitação física?^†^	Não	53,3	48,9	60,8	NS
		Sim	46,7	51,1	39,2	
11. Cognição	O idoso apresenta algum sinal de comprometimento cognitivo?^‡^	Não	44,2	41,8	52,5	<0,05
		Sim	55,8	58,2	47,5	
12. Humor	O idoso apresenta algum sinal de alteração de humor?^§^	Não	39,7	36,0	52,5	<0,01
		Sim	60,3	64,0	47,5	
13. ABVD	O idoso precisa de ajuda para alguma ABVD?^¶^	Não	82,6	83,8	78,3	NS
		Sim	17,4	16,2	21,7	
14. AIVD	O idoso precisa de ajuda para alguma AIVD?^&^	Não	31,6	30,1	36,7	NS
		Sim	68,4	69,9	63,3	
15. Incontinência	O idoso apresenta algum sinal de incontinência?^#^	Não	58,7	58,2	60,5	NS
		Sim	41,3	41,8	39,5	
16. Perda de peso não intencional	Nos últimos 12 meses o(a) Sr.(a.) perdeu peso sem ter feito dieta ou mudado qualquer hábito de vida?	Não	74,3	73,2	78,0	NS
		Sim	25,7	26,8	22,0	
17. Condições bucais	O idoso apresenta algum problema bucal ou de ingestão alimentar?^**^	Não	48,7	47,5	52,9	NS
		Sim	51,3	52,5	47,1	

^*^ Referente às seguintes condições: *diabetes mellitus*, hipertensão arterial, acidente vascular cerebral, doença arterial coronariana, doenças vasculares, lesão por pressão, anemia, asma, doença pulmonar obstrutiva crônica, úlcera péptica, artrose, obesidade, neoplasia, demência, epilepsia, depressão, doença de Parkinson, HIV/AIDS, amputação de membro, tabagismo e alcoolismo; ^†^ capacidades avaliadas: tocar a nuca, apanhar um lápis sobre a mesa com uma das mãos e colocá-lo de volta, andar 400m e sentar-se ou levanta-se sem dificuldade; ^‡^ sinais avaliados: outras pessoas falaram que os idosos está ficando esquecido, o esquecimento está piorando nos últimos meses, o esquecimento impede o idoso de realizar alguma atividade do cotidiano; ^§^ sinais presentes no último mês: desânimo, tristeza ou desesperança, perda do interesse ou prazer em atividades anteriormente prazerosas; ^¶^ atividades avaliadas: sair da cama, vestir-se, alimentar-se e tomar banho; ^&^ atividades avaliadas: atividades fora de casa e manejo de dinheiro; ^#^ incontinência: perda involuntária de urina e fezes, ^**^ condições avaliadas: prótese mal adaptada, problemas para mastigar, problemas para engolir e deixou de comer alimentos por causa de problemas nos dentes/prótese. NS: não significativo; ABVD: atividades básicas da vida diária; AIVD: atividades instrumentais da vida diária.

Pouco mais da metade da população atendida pelo PAI da CRS-Sul estava na faixa de 75 a 89 anos (53,6%), seguido pela faixa de 60 a 74 anos (42,5%). A proporção de idosos longevos (≥90 anos) foi de apenas 3,9%.

Em relação ao sexo, houve predomínio feminino (77,6%). Dentre as questões avaliadas pela AMPI/AB, destacaram-se: autoavaliação negativa de saúde (67,8% relataram ter saúde regular, ruim ou muito ruim), presença de múltiplas morbidades (50,6% relataram ter três ou mais doenças crônicas), polifarmácia (58,1% utilizavam cinco ou mais medicamentos), presença de problemas sensoriais (58,8% tinham problemas para enxergar e 41,1% problemas para ouvir), queixas de alterações cognitivas (55,8% tinham sinais de défice cognitivo), alterações de humor (60,3% tinham algum sintoma de depressão), dificuldade na realização de AIVD (68,4% necessitavam de ajuda em pelo menos uma atividade) e presença de incontinência urinária e/ou fecal (41,3%). As queixas de problemas bucais ou ingestão alimentar também foram percebidas pela metade dos idosos (51,3%), enquanto apenas 25,7% afirmaram ter perdido peso sem motivo aparente nos últimos 12 meses.

Quanto ao arranjo familiar, 25,8% dos idosos (amostra total) relataram morar sozinhos. A prevalência de quedas e de internações recorrentes (duas ou mais vezes no último ano) foi de 18,2 e 3,0%, respectivamente. A maioria dos idosos relatou não apresentar limitações funcionais que os impedissem de realizar o autocuidado (82,6%), embora 46,7% relataram apresentar alguma limitação física.

Em relação à comparação entre os sexos, foi possível observar que uma proporção maior das mulheres apresentava três ou mais doenças crônicas (54,1% *versus* 38,3%), sinais de comprometimento cognitivo (58,2% *versus* 47,5%), alterações de humor (64,0% *versus* 47,5%) e polifarmácia (62,6% *versus* 42,5%) comparativamente aos homens (p<0,05). Os demais parâmetros (idade, autoavaliação de saúde, arranjo familiar, internações, quedas, visão, limitação física, ABVD, AIVD, incontinência, perda de peso não intencional e condições bucais) não apresentaram diferenças entre os sexos quanto às suas ocorrências. Contudo, os homens apresentaram mais dificuldades em ouvir (54,6%) comparados às mulheres (37,2%).

Com relação à classificação final obtida pela AMPI/AB, a maior parte da população assistida pelo PAI era pré-frágil (45,1%) e frágil (36,1%), não sendo observadas diferenças estatísticas para a prevalência de fragilidade entre os sexos (Figura 1).

**Figura 1:** Prevalência de fragilidade em idosos assistidos pelo Programa Acompanhante de Idosos, de acordo com a classificação final da Avaliação Multidimensional da Pessoa Idosa na Atenção Básica AMPI/AB: Avaliação Multidimensional da Pessoa Idosa na Atenção Básica.

## DISCUSSÃO

A prevalência de mulheres assistidas pelo PAI foi um pouco maior (77,6%) quando comparada a de outros estudos nacionais, como o da rede Fragilidade em Idosos Brasileiros (FIBRA),^(10)^ que entrevistou 5.532 idosos de diferentes regiões do Brasil, cuja prevalência de mulheres foi de 65,6%. Da mesma forma, em estudo com dados de idosos (60 anos e mais) participantes do ELSI-Brasil,^(11)^ a prevalência de mulheres foi de 53,6%.^(3)^

A feminização da velhice, ou seja, a maior proporção de mulheres do que homens na população idosa, especialmente nas idades mais avançadas, tem repercussões importantes nas demandas por políticas públicas. Comparativamente com os homens, as mulheres tendem a conviver por mais tempo com DCNT e a ter maior risco de dependência, além de comumente assumirem o papel de cuidadoras de seus parceiros e ficarem sozinhas no final da vida.^(4,12)^ Como as mulheres também apresentam menor escolaridade e remuneração quando comparadas aos homens, as mesmas são mais suscetíveis à fragilidade e à falta de recursos financeiros na velhice.^(12,13)^ Portanto, as mulheres têm maior chance de necessitar de cuidados de longa duração, tanto pela maior incapacidade como pelo baixo suporte social, justificando a grande proporção de idosos do sexo feminino assistidos pelo PAI.

A modificação do perfil de saúde da população dos países em desenvolvimento ocorre de maneira rápida, e as DCNT passam a figurar entre as principais causas de morte e incapacidade, posições essas antigamente ocupadas pelas doenças infecto-contagiosas.^(1)^ Embora o envelhecimento não seja sinônimo de doenças, evidências acumuladas na literatura suportam a maior prevalência de DCNT com o avançar da idade.^(14)^ Esse é um fato considerável e de repercussão para as políticas públicas, uma vez que as DCNT agravam as condições de saúde, podendo resultar em declínio funcional, baixa qualidade de vida, aumento da utilização de serviços e aumento dos gastos em saúde.^(14)^

Em idosos brasileiros residentes na comunidade, a prevalência de múltiplas morbidades (duas ou mais doenças crônicas) varia de 36% a 42%.^(2,13)^ Nos idosos acompanhados pelo PAI, entretanto, a prevalência de três ou mais doenças crônicas foi de 50,6%, sendo essa maior entre as mulheres (54,1%). Dados do estudo SABE (Saúde, Bem-Estar e Envelhecimento) também corroboram a maior prevalência de DCNT nas mulheres idosas. Alvarado et al.,^(13)^ mostraram que 45,4% das idosas investigadas no estudo SABE-Brasil relataram ter duas ou mais doenças crônicas, enquanto que, para os homens, a percentagem foi de 36,4%.

Outro problema bastante comum em idosos com múltiplas morbidades diz respeito ao uso exacerbado e/ou inadequado de medicamentos. Estudos de base populacional mostram que a prevalência de polifarmácia (cinco ou mais medicamentos de uso regular) em idosos pode variar entre 10,3% e 36%,^(15-18)^ valores esses inferiores ao observado no presente estudo (58,1%). Dentre os fatores associados à polifarmácia, destacam-se a autoavaliação negativa de saúde, a presença de DCNT (principalmente do sistema cardiovascular e metabólicas) e a utilização de serviços de saúde.^(16-18)^ Adicionalmente, dados do estudo SABE^(15)^ mostraram maior risco de polifarmácia entre as mulheres e os mais velhos (>75 anos). Dentre os idosos avaliados no presente estudo, a maioria era mulher (77,6%) e grande parte tinha idade ≥75 anos (57,5%), o que pode ter contribuído para alta percentagem de polifarmácia. Foi possível observar também maior prevalência de polifarmácia entre as mulheres (62,6%) em comparação com os homens (42,5%). A polifarmácia aumenta o risco para desfechos negativos em idosos, como reações adversas, declínio funcional, declínio cognitivo, incontinência urinária, quedas, entre outros,^(19)^sendo essa prática considerada um desafio para a assistência à saúde do idoso.^(16,17)^

A autoavaliação da saúde tem sido associada ao risco de mortalidade, principalmente em idosos.^(20-22)^ De acordo com Reile et al.,^(20)^ indivíduos com autoavaliação negativa de saúde apresentam duas vezes mais risco de mortalidade em 5 anos, em comparação com aqueles que autoavaliam sua saúde como excelente, mesmo após ajustes para covariáveis importantes (comorbidades, capacidade funcional e depressão). Bamia et al.,^(21)^ também encontraram risco aumentado de mortalidade em idosos com autoavaliação negativa de saúde em diversas regiões da Europa e Estados Unidos e, portanto, concluíram que essa é uma ferramenta rápida e simples para a identificação de grupos de idosos com risco de mortalidade precoce.

Segundo Kusumastuti et al.,^(22)^a capacidade discriminativa de risco de mortalidade da autoavaliação de saúde é similar a outros índices mais objetivos (índices de comorbidade e fragilidade), reforçando a importância de considerar a percepção dos idosos quanto à sua condição de saúde.

No presente estudo, 67,8% dos idosos do PAI relataram ter saúde regular, ruim ou muito ruim, valor esse superior aos encontrados para dados populacionais de idosos residentes na comunidade. Dados da última onda do estudo SABE, por exemplo, mostraram que 48,9% dos entrevistados autoavaliaram a saúde como regular, ruim ou muito ruim,^(23,24)^ sendo que essa apresentou associação com sexo feminino, idade ≥75 anos, baixa renda e baixa escolaridade.

De acordo com Organização Mundial da Saúde (OMS), a saúde da pessoa idosa deve ser considerada a partir da perspectiva funcional, e não baseada na ausência de doenças.^(25)^A funcionalidade, por sua vez, é determinada pelo nível de ajuda que o indivíduo necessita para realizar as AVD, que é influenciada por fatores individuais (habilidades físicas e mentais) e ambientais, e pela interação entre eles.^(26)^ A instalação da incapacidade segue um processo hierárquico que, tipicamente, começa com o aparecimento de uma morbidade, que leva a uma disfunção, seguida por limitações funcionais e, em última instância, por uma determinada incapacidade.^(27,28)^ De acordo com Bleijenber et al.,^(29)^ idosos com três ou mais doenças crônicas apresentam três a cinco vezes maior risco de desenvolver incapacidade, comparativamente àqueles sem condições crônicas. Portanto, é essencial intervir precocemente no processo de incapacidade, com o intuito de preservar ao máximo a capacidade funcional de idosos em risco.

Em relação à dificuldade para realizar as AVD, foi possível observar que 17,4% dos idosos do PAI precisam de ajuda em pelo menos uma ABVD. Nesse aspecto, esse resultado corrobora o de outros estudos envolvendo idosos brasileiros, sendo que a prevalência de dificuldades em ABVD em idosos comunitários varia entre 14,8% e 25,4%.^(4)^ No entanto, foi constatado que a proporção de idosos do PAI que necessitam de auxílio para as AIVD é duas vezes maior (68,4%) quando comparada ao estudo SABE (aproximadamente 30%).^(4)^ Esses resultados sugerem maior dependência dos idosos do PAI, especialmente em tarefas mais complexas e importantes para manutenção da independência na comunidade. Este fato é preocupante, já que a capacidade de realizar atividades complexas parece declinar de maneira mais rápida em relação às atividades básicas, o que pode, a curto prazo, aumentar o grau de dependência destes idosos.^(30)^

Ao considerar a funcionalidade como principal determinante da saúde na velhice, é fundamental que a atenção ao idoso seja embasada no gerenciamento da capacidade funcional e não das doenças crônicas presentes.^(31)^ Assim, na proposta de linha de cuidado para o idoso, o risco de fragilização deve ser monitorado a partir da capacidade funcional e, consequentemente, de acordo com sua necessidade, em diferentes níveis da atenção.^(32)^ Dentro da assistência domiciliar, o PAI contribui para a atenção integral à pessoa idosa em situação de fragilidade e vulnerabilidade, uma vez que fornece apoio e suporte às necessidades de saúde e sociais.^(33)^ Nesse sentido, as diversas demandas de saúde, apresentadas pelos idosos do presente estudo, reforçam a importância do PAI na manutenção da autonomia e na independência de seus usuários.

Por fim, o presente estudo possui algumas limitações, que devem ser consideradas. Ao excluir idosos não alfabetizados, com alterações cognitivas e sensoriais, por não conseguirem responder às questões da AMPI/AB e/ou assinar o TCLE, possivelmente os mais frágeis e vulneráveis tenham ficado de fora da amostra final. Além disso, como a AMPI/AB está em processo de validação, os autores optaram por não discutir os dados relativos à classificação de fragilidade, uma vez que esse construto ainda precisa ser testado e confirmado em estudos psicométricos futuros.

## CONCLUSÃO

As piores condições de saúde de idosos vulneráveis assistidos pelo Programa Acompanhante de Idosos, constatadas pela alta prevalência de autoavaliação negativa de saúde, comprometimento das atividades instrumentais da vida diária, múltiplas morbidades, polifarmácia e outras queixas (alterações sensoriais, sintomas depressivos e cognitivas), reafirmam a importância deste programa como política de cuidados de longa duração e, portanto, para a manutenção destes idosos residindo o maior tempo possível na comunidade.
